# Municipal Hygiene

**Published:** 1884-02

**Authors:** John S. Billings

**Affiliations:** Surgeon U. S. A. (Abstract of lectures delivered in Hopkins Hall, Johns Hopkins University)


MUNICIPAL HYGIENE. THE GROWTH OF CITIES AND THEIR IMPORTANCE IN MODERN CIVILIZATION. DEATH RATE IN CITIES—COMPARISONS. THE SIGNIFICANCE OF DEATH RATES. By DR. JOHN S. BILLINGS, Surgeon U. S. A. (Abstract of lectures delivered in Hopkins Hall, Johns Hopkins University.)

1.

Dr. Billings began by defining sanitary science as a system of calculations and statements based upon probabilities, and not upon rigorously demonstrated laws. But probabilities govern a large part of the actions of men. This is an age of cities, and the tendency of the times is to congregation, association and division of labor. Statistics were adduced to show that in this country and England the urban population has been increasing at a rate far beyond the rural. The statistics of one hundred cities in the United States show an increase for ten years ending 1880 of 366 per cent., the total population of the country during the same period only increasing 30 per cent. "Speaking roundly, it may be said that in 1790, one-thirtieth of the population was found in cities of more than 8,000 population ; in 1800, one twenty-fifth ; in 1810 and 1820, one-twentieth ; in

1830, one-sixteenth; in 1840, one-twelfth; in 1850, one-eighth; in 1860, one-sixth; in 1870, more than one-fifth, and in 1880 half way between one fifth and one-quarter." (*Tenth Census, Statistics of the Population, etc., Washington*, 1881. *Page* 30.) If 4,000 people constitute a city, more than one-fourth of the population are urban. The sanitation of cities is therefore the sanitary problem of the day. The lecturer considered the various motives which should incite to effort in this direction. If other motives were lacking, he maintained that it should be done as a matter of self-protection, on the common business principle that it will pay. The annual number of deaths in Baltimore is now nearly 9,000, having been for the last two years over 2½ per cent. of the population. If we can prevent only one-tenth of this—that is reduce the rate to 2¼ per cent., or 20.5 per thousand, which certainly can be done, we shall save nine hundred lives, and get rid of one thousand eight hundred sick who are constant burden and expense. The money value of this is over a million dollars. The recent experience with small-pox cost the city treasury over $90,000, to say nothing of the cost of the deaths, and of the sickness to individuals, or the loss to the business of the city, due to the prevalence of the disease. Yet this epidemic might have been prevented at comparatively small expense. Dr. B. quoted from Ruskin and Sumner to illustrate the two different standpoints from which the question of sanitation is regarded, and said there was no real contradistinction between these apparently opposite views so far as public hygiene is concerned, so long as we are careful not to consider the latter as a matter of charity and philanthropy instead of as what it is—a matter of right and justice and of self-preservation, intended to do away with those evils which are due not so much to the struggle for existence as to the imperfections of civil institutions, and which fall chiefly on certain classes, a distinction which is very properly insisted on by Professor Sumner.

The lecturer next spoke of the improvements made of recent years in municipal sanitation, a result which had its origin about forty years ago in Great Britain, and which was largely due to vital statistics, as tabulated and expounded by Dr. Farr. The figures representing the mortality of a place are not alone the best test of its sanitary condition, but upon the whole a very good test, and often the only available one. The practical importance of accurate and complete vital statistics is not duly estimated in this country. Even health officers often give little attention to them, and are unacquainted with the proper method of using them. The most fruitful cause of error in calculating mortality rates is connected with the mode of estimating the average living population which has furnished the deaths for a particular period. In a rapidly growing city, with a fluctuating population, it is very difficult by any method to make reliable estimates as to the number of living population for any other year than that in which a complete census is taken. To illustrate some of the methods employed and their different results, the lecturer referred to the case of Baltimore. The population of this city, according to the United States census of 1880 was 332,313.

UNITED STATES CENSUS, 1870.

| | |
|---|---|
| Total population | 267,354 |
| Number of families | 49,929 |
| Persons to a family | 5.35 |
| Number of dwellings | 40,350 |
| Persons to a dwelling | 6.63 |

UNITED STATES CENSUS, 1880.

| | |
|---|---|
| Total population | 332,313 |
| Number of families | 65,356 |
| Persons to a family | 5.08 |
| Number of dwellings | 50,883 |
| Persons to a dwelling | 6.54 |

The method ordinarily used by statisticians is to suppose that the population has increased since 1880 in the same proportion as between 1870 and 1880: thus estimated, the population of the city, June 1, 1881, would have been 339,620 ; June 1, 1882, 347,087 ; June 1, 1883, 354,720. These figures are believed by the Health Officer of Baltimore and by the gentleman charged with the registration of its vital statistics to be too small, and they prefer to take as the basis of their calculation the number of voters, as ascertained by the police census, and to assume that each voter represents five persons of the population. In this way the population is calculated at the middle of 1882, as 408.520. The city health authorities refuse to accept the census of 1880, and estimate the population at that time to have been 393,796, or over 61,000 more than the census, a deficiency in the latter, according to them, of over 15 per cent. Their estimate is based upon the following considerations:

" There are 80,000 registered legal voters in Baltimore; five inhabitants to a legal voter is a fair and reasonable allowance, which would make the population 400,000. The census taken by the police for our School Board, of children between the ages of six and twenty-one years, gave 86,961. This would be a fair estimate of one-fifth of our population, making the same 434,805. * * * Again, there are 90,000 houses in Baltimore. Deduct 10,000 for manufacturing establishments, warehouses, stores, and unoccupied dwellings, estimating five inhabitants to each house (a very low estimate) our population would be 400,000." (A. R. Carter, letter in San Engineer, Feb. 15, 1881, p. 130). The lecturer said: " A failure to enumerate 15 per cent., or more than one out of every seven inhabitants could only result from criminal negligence on the part of the enumerators, a negligence directly opposed to their pecuniary interests." General Walker says of the census of Baltimore that it was taken by a thoroughly capable and experienced officer, who had had experience in the same field in 1870; that he knew of nothing impeaching the completeness and integrity of this officer's work; that the danger of omission in a city like Baltimore, with comparatively few persons in a dwelling or tenement house was at a minimum; that the children between six and twenty-one years of age constitute probably between one-third and two-fifths rather than one-fifth, a proportion only found in Paris, " where the science of preventing infant life is very thoroughly understood and extensively practiced; " that if the ratios of New York, Brooklyn or Albany, were applied to the school population of Baltimore as determined by the police census, the inhabitants would be less than 290,000; even according to the ratio of Boston, where the proportion of school children to population is less than in any other American city, the population would still be less than 316,000.

Dr. B. then pointed out that the police census shows a diminution rather than an increase of the voters. Also that the number five is much too large a ratio ; the ratio should be calculated, rom census data, not assumed. In Boston, where the estimate is made in the same way (owing to a recent extension of the city limits), four is taken as the ratio, which is evidently much more nearly correct. The truc ratio for Baltimore is about 4.25. The estimate as to the number of houses is much too great, and the lecturer saw no reason to doubt the accuracy of the census figures. Their correctness is confirmed by the police census of houses taken in December, 1883, which gave the following results: Number of dwellings, 54,546 ; of places of business, 6,885 ; of churches, 267 ; total, 61,698. This corresponded with data which the lecturer had obtained from the Water Department, according to which, on January 1, 1883, there were 51,284 houses supplied with water and 6,000 unsupplied. "From these data I think we may conclude that there are not over 54,500 inhabited dwellings at present within the city limits, and if we allow to each 6.50 persons, which is the ratio shown by the last census, we should obtain 356,430 as the present number of inhabitants. If we take the last police census and, making a little allowance for those at that time absent from the city, take the number of voters at 82,000, and take the ratio at 4.03, which is larger than that derived from the census data, we find that the number of inhabitants is 352,600. Taking all these data together, the probability becomes very great that the living population within the city limits on January 1, 1883, was between 350,000 and 356,000 persons." "The mode of computing population by the number of occupied houses is one which gives fairly accurate results, provided the number of such houses is accurately ascertained, but it will not do to merely estimate them."

None of our cities possess accurate birth statistics, except possibly a few in Massachusetts ; the deaths are, however, in most cities, fully and fairly reported.

The lecturer then proceeded to show the influence of cities on the health of their inhabitants, and showed that the mortality is greater in large cities than in small, and greater in the latter than in rural districts. The lecturer next showed a diagram giving the death rate in Baltimore for each of the last thirty-three years. The average for the ten years preceding 1860 was higher than since, but it now seems to be slowly increasing. The diagram gives the impression of large waves of variation, with a period of from ten to fifteen years, and the surfaces of these large waves are irregular from the smaller variations for the single years. These great weves may possibly be connected with changes in the sun, shown by the sun-spots.—*Maryland Med. Journal.*

---

TREATMENT OF WARTS WITH MAGNESIA.

Lucas-Championnière reports in the *Journal de Méd. et de chir. prat.*, August, 1883, a case of a young child, aged 8 years, cured of a great number of warts on both hands by the treatment of Foussagrives. This treatment consists in giving each day 80 centigr. of calcined magnesia internally. The cure was complete in two months, and due to the action of the magnesia. The author remembers the anatomical analogies existing between warts and epitholiomas, and thinks that the magnesia may give equally good results in the latter. It will be interesting to make this research.—(*La France Médicale.*)

RUMINATION AMONG THE INSANE.

Dr. Bouchard read a paper at the Society of Medical Sciences of Lille, on the rumination, which known as a pathological rarity, is in reality very frequent, especially in insane asylums. There are fourteen ruminating patients in the asylum of Lommelet; eleven among 100 idiots, and three among 576 patients. Many of these patients ruminate before presenting any symptoms of insanity; therefore this symptom might have a grave diagnostic significance.—*Journal des Sciences Méd. de Lille.*

